# Selenium Differentially Influences Methylmercury Retention across Mayfly Life Stages

**DOI:** 10.1021/acs.est.5c00338

**Published:** 2025-04-16

**Authors:** Jacqueline R Gerson, Rebecca Dorman, Collin Eagles-Smith, David M. Walters

**Affiliations:** a Cornell University, 111 Wing Dr, Ithaca, New York 14852, United States; b U.S. Geological Survey, 4200 E New Haven Rd, Columbia, Missouri 65201, United States; c U.S. Geological Survey, 777 NW ninth St, Corvallis, Oregon 97330, United States

**Keywords:** life history, insect metamorphosis, mayfly, mercury−selenium interactions, trophic transfer

## Abstract

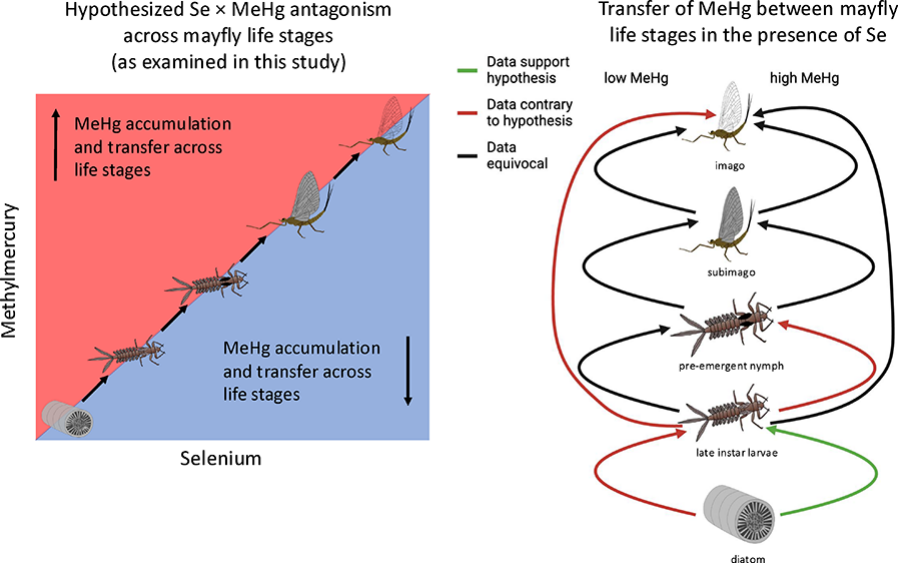

Though high mercury and selenium concentrations are individually toxic to organisms, there is a hypothesized antagonistic relationship. This potential mercury–selenium interaction is under-studied in aquatic macroinvertebrates, particularly in relation to complex life histories. We examined the proposed effect of selenium on methylmercury accumulation between four life stages for a parthenogenetic mayfly (*Neocloeon triangulifer*). We exposed diatoms to elevated methylmercury concentrations and fed them to mayflies exposed to elevated aqueous selenomethionine. We found some support for the mercury–selenium antagonism hypothesis, but it was context-specific. Selenium reduced methylmercury accumulation in high but not low methylmercury environments. Though terrestrial adult life stages had higher mercury concentrations compared to aquatic larval life stages, cumulative life history transfer factor (LHTF; ratio of methylmercury in adult imago to late instar larvae) differed by treatment. LHTF was constant for all aqueous selenium exposure levels at high dietary methylmercury (selenium impacts on methylmercury uptake and loss) but increased with aqueous selenium exposures at low dietary methylmercury (selenium impacts on methylmercury uptake only), suggesting a synergistic enhancement of MeHg transfer between life stages with increased aqueous Se exposure levels. These results suggest that animals eating adult aquatic insects are exposed to higher concentrations of methylmercury than those feeding on larval insects across selenium and methylmercury levels, but interference of selenium on methylmercury accumulation is only present at high methylmercury environments.

## Abstract 2

Support for the selenium antagonism of mercury hypothesis is context-dependent; selenium reduces aquatic insect methylmercury accumulation under certain conditions but can also enhance methylmercury transfer from larvae to adults.

## Introduction

Mercury (Hg) in the form of methylmercury (MeHg) is a potent neurotoxin that can negatively impact the growth, development, behavior, and survival of organisms.^1,2^ Organic forms of selenium (Se), while important as micronutrients, can also be toxic to organisms at high levels, leading to teratogenic effects and reproductive harm.^3−6^ However, some evidence suggests that in certain circumstances, Se may be protective of MeHg toxicity and accumulation in organisms.^7−9^ This “Se antagonism hypothesis” is the basis for the argument that environmental Se lowers the risk of Hg exposure to consumers and that Se can be incorporated into risk management strategies. Examples of these strategies include calls to alter fish consumption advisories to reflect Se:Hg ratios^10^ and to remediate Hg-contaminated ecosystems with Se additions.^9,11^ However, a recent literature review found limited and ambiguous support for the Se antagonism hypothesis – with some studies reporting synergistic rather than antagonistic effects of Se on Hg – indicating that such risk management actions are premature and could pose additional risks to organisms.^9^

Gerson et al.^9^ considered three specific hypotheses related to Hg–Se interactions: “(1) dietary Se reduces MeHg toxicity in consumers, and/or (2) environmental Se reduces Hg bioaccumulation and biomagnification in aquatic food webs, and/or (3) Se inhibits Hg bioavailability to, or MeHg production by, microbial” communities that methylate Hg. Different mechanisms have been proposed for each of these hypotheses, but these mechanisms driving the hypothesized Hg–Se interactions remain poorly constrained. The first hypothesis has garnered much attention in the literature, though results of studies to date are ambiguous. In contrast, fewer studies have examined the second hypothesis focused on the interaction of Hg and Se on bioaccumulation of each element in aquatic macroinvertebrates such as insects.^9,12^ Aquatic insects play a central role in the flux of energy and materials within aquatic food webs and between aquatic and terrestrial food webs,^13,14^ so it is vital to understand how tissue concentrations of these co-occurring elements might vary over their complex life cycle. The third hypothesis has also received little attention in the literature and warrants further study, which is beyond the scope of this paper.

Aquatic Hg and Se enter aquatic food webs after the production of organic forms (i.e., MeHg, selenomethionine; SeMet) by primary producers such as diatoms. This process leads to enrichment factors up to 6 orders of magnitude above aqueous concentrations, before being consumed by aquatic invertebrates and other animals.^15−18^ Methyl Hg and Se can then be transferred to terrestrial ecosystems via aquatic insects. Aquatic insects have an aquatic larval stage but emerge from the water as flying adults, serving as a vector of aquatic-terrestrial energy and contaminant flux.^14,19−22^ For example, previous studies have shown that Hg and Se concentrations in riparian spiders are well correlated with Hg and Se fluxes associated with adult aquatic insects.^21,23^ This transfer of MeHg and Se from aquatic to terrestrial food webs can result in elevated concentrations in other riparian animals that feed on aquatic-derived prey, like songbirds.^24,25^ During the various life stages and life transitions of insects, contaminants may be shed (via loss of exuvia, excretion, or both) or conserved and bioamplified, depending on the contaminant’s chemical properties and tissue distribution in organisms.^19^ Contaminant retention versus loss has important implications for its availability to aquatic or terrestrial predators that eat aquatic insects.^26−28^ However, there is little information about how complex life histories, such as those with metamorphosis, affect MeHg retention, accumulation, and transfer in the presence of Se.

In this study, we exposed larval mayflies (*Neocloeon triangulifer*) to two dietary levels of MeHg (low and high) in their food and to a gradient of SeMet concentrations in the water column. We used this controlled dosing experiment to ask: (1) Does Se affect MeHg bioaccumulation in mayflies? (2) Does the effect of Se on MeHg bioaccumulation in mayflies vary by life stage and/or magnitude of MeHg exposure? (3) Does Se influence the retention of MeHg between life stages? If Se is antagonistic to MeHg at the base of the food web, we would expect increasing Se exposure levels to reduce retention of MeHg. We therefore would expect MeHg concentrations and transfer factors to decrease across life stages as aqueous Se exposure levels increase. We would also expect that this pattern would hold regardless of dietary MeHg exposure level. Addressing these questions and hypotheses is important to evaluate the extent of Se and MeHg interaction at the base of the food web, with implications for when these elements co-occur in the environment as well as for management practices.

## Materials and Methods

### Experiments

We conducted dosing experiments at the U.S. Geological Survey (USGS) Columbia Environmental Research Center (CERC), USA. Specific details on the experimental conditions can be found in Gerson et al.^29^ A brief summary is provided here. We obtained genetically identical eggs from the parthenogenetic mayfly *N. triangulifer* (Stroud Water Research Center Clone White Clay Creek 2; #WCC-2). *N. triangulifer* is often used as a model organism in ecotoxicological and physiological studies since they are genetically identical, have a short life span (∼28 d), can easily be reared in the laboratory, and allow for an assessment of impacts on sensitive insect orders representative of those found in natural streams.^30−32^*N. triangulifer* eggs are deposited in water bodies and hatch into an aquatic larval stage. During this instar larval stage, they feed on diatoms and receive contaminants via dietary input.^18,33−35^ They then transform into a nonfeeding terrestrial stage as winged adults, during which they can transfer contaminants from aquatic to terrestrial ecosystems.

We cultured diatoms and mayflies in environmental chambers according to Soucek and Dickinson.^31^ Mayflies spent their first 12 days in an autoclaved beaker with 1 L of Duluth 100 water^36^ and one diatom slide, with a 1L water change and diatom slide change on day 7. After growing mayflies in Duluth 100 water for 12 days, we transferred 35–45 larvae to each exposure tank containing 4 L of clean Duluth 100 water with continuous aeration and a range of selenomethionine (SeMet) concentrations (2 exposure tanks for each of the 7 aqueous SeMet exposure levels; see below for more information) and fed the mayflies diatoms grown on slides that had been exposed to elevated levels of MeHg (low and high).

Prior to the mayfly exposure experiments, diatom slides were placed in polyethylene terephthalate copolyester glycol (PETG) containers containing 5 L of water with low (0.2 ng MeHg/L) or high (2 ng MeHg/L) MeHg concentrations for at least 48 h. We selected this concentration range to span an order of magnitude, based on the range of MeHg found in the environment, from natural to artisanal and small-scale gold mine-impacted water bodies (∼0–20 ng/L MeHg)^37^ and based on common concentrations in freshwater invertebrates (10–100 ppb).^38^ Diatoms accumulated an average of 4 ± 2 ng MeHg/g dw in the low MeHg treatment and an average of 9 ± 5 ng MeHg/g dw in the high MeHg treatment, with variability likely due to diatom quality and duration of diatom MeHg exposure.^37^ We provided mayflies with two MeHg-exposed diatom slides at the beginning of the experiment, and we replaced the slides when >50% of the diatom slides had been grazed, as determined by visual assessment.

We performed the experiments using SeMet exposure to simulate the form of Se to which organisms are exposed during bioaccumulation. While the predominant form of Se in water and sediment is inorganic, organo-Se (e.g., SeMet) is the form that is toxic,^5,39,40^ bioaccumulative,^17^ and potentially protective of MeHg.^41−44^ Biofilms take up inorganic Se from the water column and convert it into organo-Se, and mayflies are generally exposed to organo-Se through their diet. For this reason, SeMet has been identified as a good proxy for natural dietary organo-Se.^39^ We used SeMet due to the short lifespan of mayflies and uncertainty surrounding the kinetics of Se-Met formation by diatoms and the associated microbial community. SeMet was administered in the aqueous form while MeHg was administered in the dietary form to ensure that we were measuring interaction between Se and Hg during mayfly accumulation, as opposed to biofilm accumulation. This ensured that we explicitly tested the interaction of MeHg with the form of Se that bioaccumulates. However, since we used SeMet in these experiments, the toxicological responses of mayflies to Se may be a result of exposure to aqueous SeMet, dietary SeMet, or a combination; it is not possible to determine the mechanism of toxicity based upon this study design. We prepared all SeMet and MeHg solutions from stock solutions of seleno-l-methionine (Sigma-Aldrich) and MeHg (10 ppm; Brooks Rand). Note that we analyzed samples for total Se (TSe), with the assumption that all Se present was in the form of SeMet that we prepared in the laboratory.

We conducted the mayfly experiment at seven aqueous SeMet exposure levels for each of the low (0.2 ng MeHg/L) and high (2 ng MeHg/L) MeHg treatments. We selected this concentration range based on the range of total Se found in the environment, from natural to mountaintop mine-impacted freshwater bodies (∼0–60 μg/L total Se),^45^ with SeMet representing an unknown fraction of the total Se. Our goal was to measure the interaction between SeMet and MeHg across the broadest range possible. Based on data reported in Gerson et al.,^12^ we used concentrations of SeMet below 3.2 μg total Se/L, as higher concentrations were lethal to mayfly larvae.^12^ Measured aqueous Se concentrations were 0.22, 0.53, 0.64, 0.99, 1.9, 2.29, and 3.07 μg total Se/L at low MeHg and 0.33, 0.37, 0.47, 1.35, 1.85, 2.38, and 3.1 μg total Se/L at high MeHg within 14 aquaria (9.46 L tank with 4 L of water).

We sampled mayflies at four life stages: late instar larvae, pre-emergent nymph (PEN), subimago, and imago. We defined late instar larvae as the life stage immediately prior to the development of wingpads, PEN as the larval life stage occurring upon development of wingpads and immediately prior to metamorphosis and emergence from water (lasting for roughly 24 h),^31^ subimago as the first adult life stage (lasting for roughly 12 h), and imago as the life stage after a final molt and immediately prior to egg-laying. We composited at least six mayflies by life stage and treatment tank to obtain sufficient mass for MeHg analysis. Note that not all mayflies per life stage were collected on the same date, as growth rates differed by treatment.

We assessed MeHg bioaccumulation in mayflies by two methods: the absolute concentration of MeHg in mayfly tissue and the MeHg burden accumulation (calculated as the ratio of MeHg concentration in mayflies to diatoms).^46^ The MeHg concentration in diatoms at various MeHg exposure levels and SeMet treatments is reported in Gerson et al.^12^ Notably, MeHg concentrations in diatoms across SeMet treatments was constant for the high MeHg treatment but showed a linear decrease in the low MeHg treatment (Figure S1). Thus, we used a constant diatom MeHg concentration to calculate mayfly MeHg burden accumulations for the high MeHg treatment, and we modeled diatom MeHg concentrations to calculate mayfly MeHg burden accumulations for the low MeHg treatment, as described in.^12^

We collected six to 18 mayflies from each life stage in glass vials using plastic forceps. We double-bagged the vials and stored them frozen until lyophilization of the composite mayflies by life stage and treatment. We digested lyophilized mayflies in 30% nitric acid at 60°C overnight. We analyzed mayflies for MeHg concentration with a Brooks Rand Merx-M (Seattle, WA, USA) automated MeHg analyzer (EPA Method 1630). Standard recoveries were 97.8 ± 1.0% (mean ± SE) for certified reference materials (n = 27; IAEA407: fish homogenate; IAEA452: scallops), 98.0 ± 1.8% for liquid calibration standards (n = 12), and 102.4 ± 2.1% for matrix spikes (n = 6). Duplicate relative percent differences were 1.7 ± 0.5 (n = 7). Digest blanks were 0.023 ± 0.022 pg MeHg (n = 12), and reagent blanks were 0.104 ± 0.012 pg MeHg (n = 12).

We measured all collected mayflies for length. We used photographs taken on 1 mm grid paper and uploaded to ImageJ to determine the distance from the anterior head capsule to the posterior abdomen, which we defined as mayfly length. We calibrated and performed at least one quality control verification for each individual photograph. All quality control measurements were within 5% of the value.

### Chemical Analyses

We collected water samples for MeHg analysis in new 125 mL PETG bottles and acidified to 0.4% with hydrochloric acid. We double bagged the bottles and stored them at 4°C until analysis. We analyzed water samples for MeHg concentration using aqueous ethylation with sodium tetraethylborate, purge and trap, and cold vapor atomic fluorescence spectrometry (CVAFS) paired with inductively coupled plasma mass spectrometry (ICP-MS; EPA Method 1630).^47^ All standard recoveries were within 15% of accepted values.

We collected water samples for TSe by filtering the water through a 0.45 μm poly(ether sulfone) (PES) filter into an acid washed 60 mL high density polyethylene (HDPE) bottle and then storing the sample at −18 °C until analysis. We analyzed water samples for TSe with a PerkinElmer Elan DRCII ICP-MS. All standard recoveries were within 10% of accepted values, and the detection limit was 0.3 μg TSe/L. Other water quality data are reported in Gerson et al.^12^

### Statistical Analyses

We performed all statistical analyses using RStudio version 2023.06.2 + 561 and R version 4.2.2,^48^ and all figures were made using the ggplot2 package.^49^ We first compared MeHg concentrations and mayfly MeHg burden accumulations across life stages using ANCOVA with interactions (aqueous Se exposure level, life stage) to determine whether Se influenced MeHg or its mayfly MeHg burden accumulation differently among life stages. As interactions were not present, we then modeled mayfly MeHg accumulation using linear regression for the low and high dietary MeHg treatments, with aqueous Se exposure as the independent variable and either mayfly MeHg concentration or mayfly MeHg burden accumulation as dependent variables. All raw data and associated metadata are available online.^29^

Ratios are commonly used to describe important step changes in contaminant uptake in organisms (e.g., biomagnification factors). Here we use ratios to assess the proportional accumulation of MeHg between life stages for each MeHg dietary treatment and Se exposure level, as well as the overall effect of these life history changes across the Se gradient: (1) elimination transfer factor: PEN MeHg concentration compared to late instar larvae; (2) metamorphic transfer factor: subimago MeHg concentration compared to PEN (following Kraus et al.^50^); (3) molting transfer factor: imago MeHg concentration compared to subimago; and (4) cumulative life history transfer factor (LHTF): imago MeHg concentration compared to late instar larvae. The elimination transfer factor can be related to a number of factors including cessation of eating and egestion of MeHg contaminated diatoms or excretion of internalized MeHg prior to metamorphosis. It is important to recognize that concentrations in PEN can be higher than larvae (which would indicate a gain rather than elimination) owing to proportional differences in body mass loss relative to elimination during this life transition. We modeled each transfer factor using linear regressions at low and high dietary MeHg treatments. We compared transfer factors between low and high dietary MeHg treatments using an ANOVA followed by Tukey’s posthoc analysis.

## Results and Discussion

### MeHg Accumulation in Mayflies

**Figure 1:**
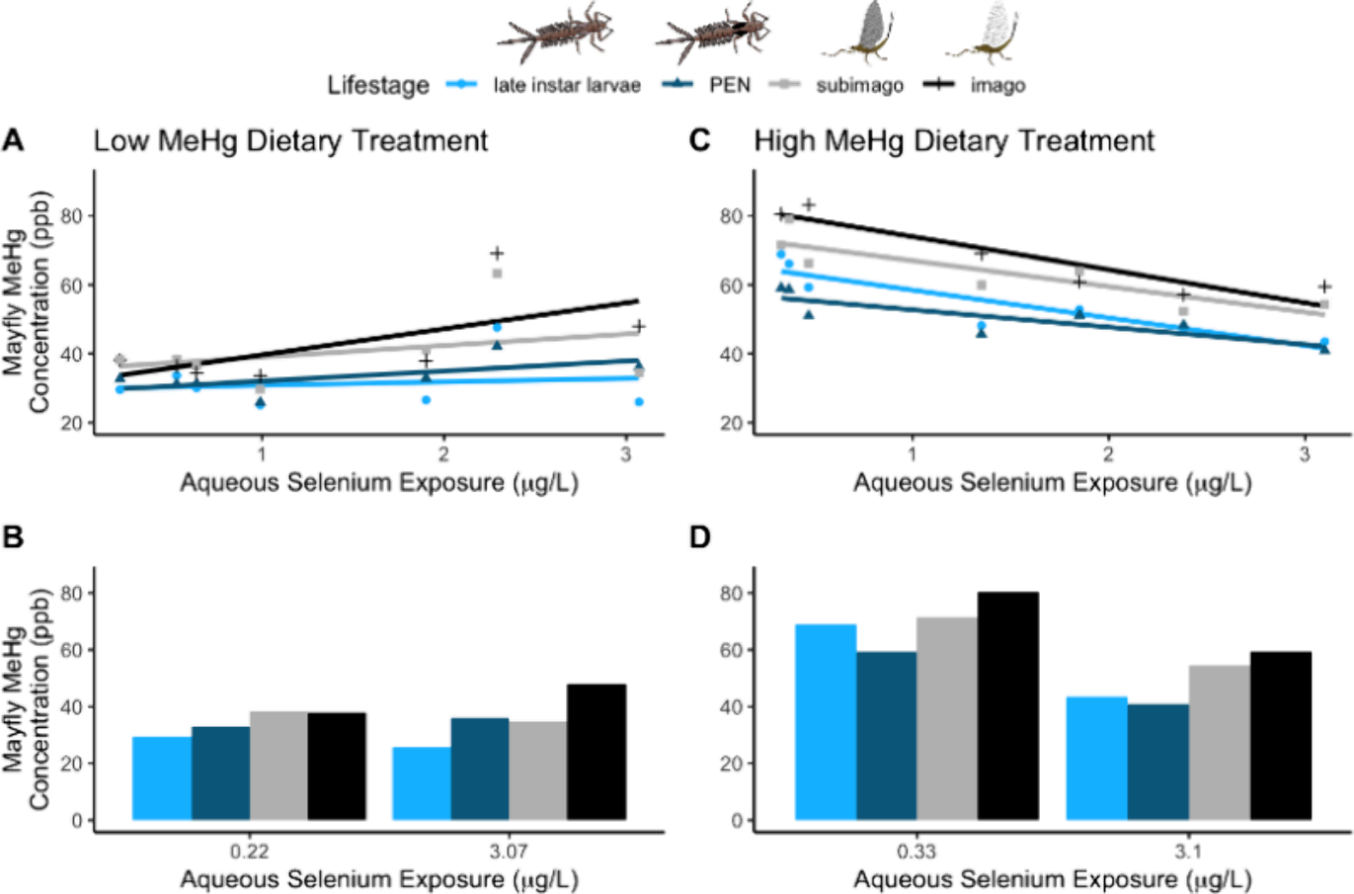
Accumulation of methyl mercury (MeHg) in mayflies across varying aqueous selenium (Se) exposures at low and high dietary MeHg treatments. Panels A and C include all measured data; light blue circles show data for late instar larvae, dark blue triangles show data for pre-emergent nymph (PEN), light gray squares show data for subimago, and black crosses show data for imago. Panels B and D show the mayfly MeHg concentrations at the highest and lowest measured Se exposure concentrations for each life stage. Points are composite values (six to 18 mayflies) by Se exposure concentration. Linear regression lines are fit for each life stage from the full ANCOVA model, though note that the model found no statistically significant differences between life stages.

Methylmercury concentrations in adult terrestrial life stages were higher than in aquatic life stages across aqueous Se exposure levels and MeHg treatments (*p* < 0.00001, F(1,3)=72.5 for high MeHg; *p* = 0.041, F(1,3)=4.69 for low MeHg; Figure 1). This suggests that MeHg is retained and concentrated across metamorphosis, similar to findings by Buckland-Nicks et al. in dragonflies (Odonata: Anisoptera).^51^ Thus, predators of terrestrial mayfly life stages are exposed to higher MeHg concentrations compared to those of aquatic mayfly life stages. However, the higher dietary MeHg treatments resulted in tissue MeHg concentrations that were almost double those in mayflies from lower dietary MeHg treatments at low Se exposure – a trend that was consistent across life stage. This contrasts with other studies that found lower transfer of metals, including Hg, from higher exposure environments.^50^ Our findings suggest that dietary MeHg treatment level, as determined by the concentration of MeHg present in water and thus biofilms, has an overwhelming influence on the accumulation of MeHg within adult and larval mayfly life stages.

### Effect of Se on MeHg Accumulation

Importantly, there was context-dependent support for elevated Se concentrations reducing MeHg accumulation in mayflies. There was limited evidence of a relationship between larval mayfly MeHg accumulation and aqueous Se exposure at low dietary MeHg treatments (*p* = 0.058, F(1,26)=3.9; [mayfly MeHg] = 3.7 * [aqueous Se] + 31.7; Figure 1A,B). However, MeHg concentrations and mayfly MeHg burden accumulations were negatively correlated with aqueous Se exposure in the high dietary MeHg treatments (*p* = 0.00018, F(1,26)=19.1; [mayfly MeHg] = −7.9 * [aqueous Se] + 71.6; Figure 1C,D, Figure S1D). Consequently, tissue MeHg concentrations for both the low and high MeHg treatments were similar at the highest aqueous Se exposure level, despite nearly a 2-fold difference at low aqueous Se exposure.

Mayfly MeHg burden accumulations increased across aqueous Se exposure levels for low dietary MeHg treatments (*p* < 0.00001, F(1,3)=48.5; Figure S1B), likely driven by patterns in diatom MeHg concentration which decreased with increased aqueous Se exposure (*p* = 0.0031, F(1,13)=13.11; Figure S1A). While the higher dietary MeHg treatments resulted in mayfly MeHg burden accumulations that were almost double those in mayflies from lower dietary MeHg treatments (and dependent on a constant diatom MeHg concentration with increased aqueous Se exposure; *p* = 0.30; Figure S1C), this pattern was reversed at high Se exposure when the lower dietary MeHg treatments exhibited nearly double mayfly MeHg burden accumulations (Figure S1B,D). This trend was consistent across life stages. Thus, our results provide partial support for the hypothesis of antagonistic protection of Se against MeHg bioaccumulation and burden accumulation,^9^ but only at high dietary Hg exposure.

### MeHg Transfer between Life Stages

**Figure 2:**
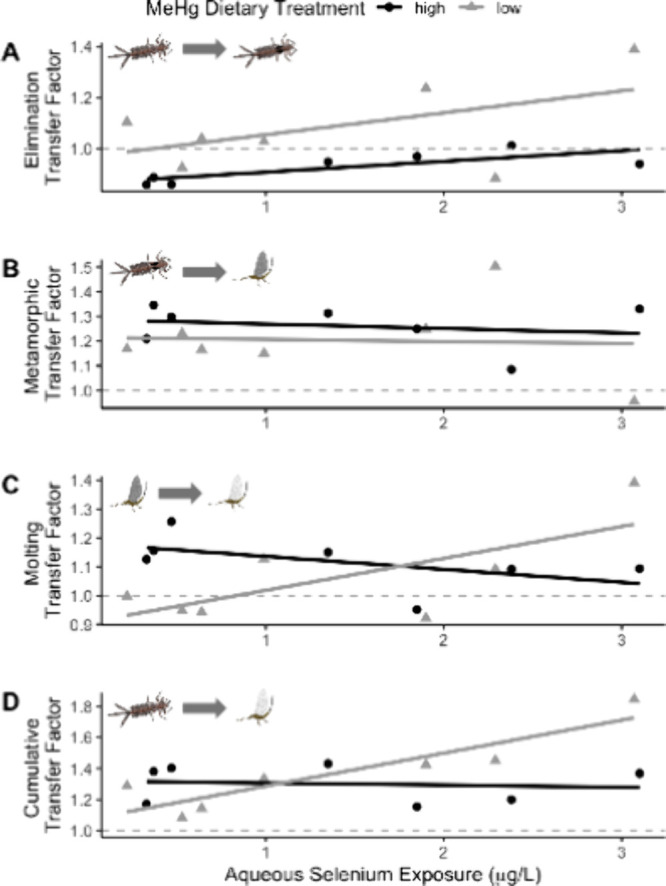
Transfer factors of methylmercury (MeHg) between mayfly life stages at low and high dietary MeHg treatments. A) Elimination transfer factor, which represents the ratio of mayfly MeHg concentrations between the pre-emergent nymph (PEN) and late instar larvae life stages. B) Metamorphic transfer factor, which represents the ratio of mayfly MeHg concentrations between the subimago and PEN life stages. C) Molting transfer factor, which represents the ratio of mayfly MeHg concentrations between the imago and subimago life stages. D) Cumulative life history transfer factor (LHTF), which represents the ratio of mayfly MeHg concentrations between the imago and late instar larvae life stages. Points are composite values (six to 18 mayflies) by Se exposure concentration. Linear regression models are fit for low (gray lines) and high (black lines) MeHg treatments. The dotted line shows a transfer factor of 1, which represents the boundary between net retention and net loss of MeHg across life stages.

Contaminant losses between life stages result from physiological processes, such as depuration (elimination), meconium excretion during formation of adult tissue (metamorphosis), and shedding of exuviae (molting).^19,52^ We calculated the ratios of MeHg concentrations across these different processes to determine the transfer of MeHg from one life stage to another across aqueous Se exposure levels and MeHg treatments (Figure 2). A transfer factor >1 indicates that net amount of MeHg relative to body tissue mass is retained across life stages, whereas a transfer factor <1 indicates MeHg loss between life stages. Predicted aqueous Se exposure levels above which the transfer factor is equal to 1, as calcuated by linear regression for the low and high MeHg dietary treatments, and are provided in Table S1.

Irrespective of Se exposure, MeHg was differentially retained between MeHg treatment levels during the elimination phase between larval and PEN stages (*p* = 0.029). At low MeHg treatments, there was a catabolic increase in MeHg concentrations (elimination transfer factor >1; mean = 1.1 ± 0.2 (sd); Figure 2A) as biomass was lost. In contrast, at high MeHg treatments, a proportionally larger share of MeHg was eliminated and lost (elimination transfer factor <1; mean = 0.93 ± 0.06 (sd); Figure 2A), presumably as MeHg-rich diatoms were egested. These results suggest that egestion and other aspects of elimination leading up to metamorphosis may be an important methodological step to consider when determining MeHg concentrations in mayflies, which is contrary to findings for other food web tracers such as carbon and nitrogen isotopes.^53^

The metamorphic transfer factor typically exceeded 1 across aqueous Se exposure levels (Figure 2B), while the molting transfer factor exceeded 1 for high MeHg treatments but were more variable for low MeHg treatments (half <1 and half >1; Figure 2C). These results collectively suggest catabolic increases in MeHg concentrations across the life stages. As mayflies transitioned to subimago and sometimes during the transition to imago, they lost mass but retained MeHg, suggesting that shedding of MeHg in exuvia is minimal and insufficient to offset loss of biomass during these transitions.^19^ Instead, each subsequent life stage generally exhibited a small increase in MeHg concentration. The largest transfer factors occurred during metamorphosis (∼1.2), suggesting that the aquatic to terrestrial transition had an outsized impact on the cumulative life history transfer factor (LHTF; Figure 2D). These findings contrast Buckland-Nicks et al.,^51^ which reports no change in MeHg concentrations between naiad, emerging adult, and mature adult dragonflies. Thus, the transfer of MeHg between life stages appears to be taxa-dependent for aquatic insects.

### Sublethal Effects of Aqueous Se Exposure

**Figure 3:**
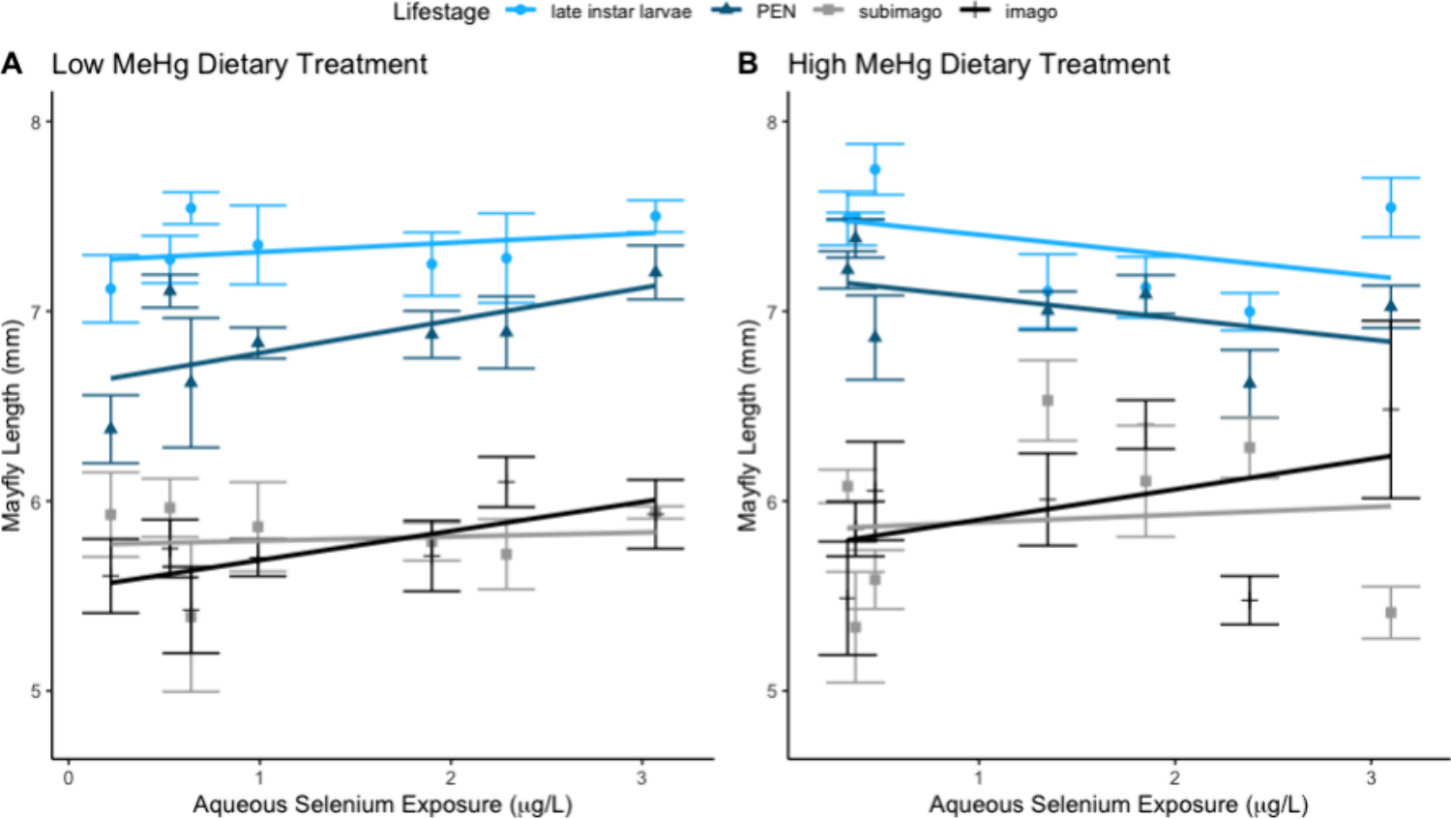
Average mayfly length compared to aqueous selenium (Se) exposure. Lines shown are the modeled linear regressions for the four life stages (late instar larvae, pre-emergent nymph [PEN], and subimago, and imago) at A) low and B) high methyl mercury (MeHg) exposure concentrations. Points are composite values (six to 18 mayflies) by Se exposure concentration. Error bars show 95% confidence intervals. Linear regression lines are fit for each life stage from full ANCOVA model.

Mayfly length was positively correlated in most life stages, with aqueous Se exposure increases associated with mayfly length in most life stages at the low dietary MeHg treatment, but negatively correlated at high dietary MeHg treatment. At the low dietary Hg treatment, body length differed among life stages (*p* < 0.00001), with late instar larvae > pre-emergent nymph > subimago = imago (*p* < 0.0002; Figure 3). This pattern is consistent with expectations, as PEN do not consume food and molting occurs between the other life stages. There was only a moderate increase in mayfly length with Se exposure level (*p* = 0.078), and only a moderate difference in rate of length increase across Se exposure levels between the life stages (*p* = 0.070). For all life stages except subimago, higher aqueous Se exposure level was associated with larger mayfly length (i.e., positive slopes).

At the high dietary MeHg treatment, body length differed among life stages (*p* < 0.0001), as did the relationship between length and aqueous Se exposure level (*p* = 0.043), with late instar larvae > pre-emergent nymph > subimago = imago (*p* < 0.027). However, there was only a moderate influence of Se exposure level on mayfly length (*p* = 0.079); higher Se exposure levels at the high dietary Hg treatment were associated with smaller pre-emergence lengths (i.e., negative slopes), but larger post-emergence lengths (i.e., positive slope for imago, no change for subimago). Note that for all the life stages and exposure levels, the size difference is very small (<1 mm) and likely ecologically irrelevant, suggesting that the presence of Se does not impact sublethal effects across mayfly life stages.

### Effect of Se on MeHg Transfer between Life Stages

We found evidence for a synergistic increase in MeHg transfer between life stages associated with Se exposure – rather than the hypothesized antagonistic relationship^9^ – though the effect differed by MeHg treatment level. At low dietary MeHg treatment, we found that higher aqueous Se exposure led to increased transfer factors across mayfly cumulative life history (i.e., positive slope; *p* = 0.0063, *r*^2^=0.76; Figure 2D), despite Se exposure not impacting any of the individual transfer factors (i.e., elimination, molting, and metamorphosis; *p* > 0.05). This effect was likely caused by greater MeHg uptake by mayfly larvae at higher aqueous Se exposure levels (Figure 1A), supporting the hypothesis that mixtures of contaminants in an ecosystem have the potential for synergistic enhancement of bioaccumulation.^54^ A synergistic relationship between MeHg and Se has previously been demonstrated for growth, reproductive, metabolic, and toxicity end points,^55−59^ and the results from this study further show the potential effects of synergism between these two elements. In contrast, at high dietary MeHg treatment, increasing aqueous Se exposures only led to larger MeHg transfer factors during the elimination phase from larval to PEN stages (*p* = 0.036, *r*^2^=0.54; Figure 2A).

The cumulative life history transfer factor (LHTF; i.e., transfer from late instar larvae to imago; ∼ 1.1–1.8 between treatments) appears to be driven primarily by the molting transfer factor. At low dietary MeHg treatments, higher aqueous Se exposure levels led to increased MeHg transfer across life stages (*p* = 0.0063; Figure 2D), likely due to retention of proportionally more MeHg at this final molting stage to imago. These results suggest that, contrary to our hypothesis, at low dietary MeHg treatments, MeHg concentrations in emergent mayflies – representative of MeHg that would be transferred to terrestrial predators – increase as aqueous Se exposure increases. In contrast, at high dietary MeHg treatments, aqueous Se exposure levels did not affect MeHg transfer across life stages (*p* = 0.79), likely due to an additional loss of MeHg at the molting stage from shedding the exuvia.

Many previous studies have examined the retention and loss of various contaminants during metamorphosis in insects. Metals are typically lost during this process, whereas highly lipophilic organic compounds like polychlorinated biphenyls (PCBs) and some pesticides are often retained and sometimes concentrated through body mass loss during metamorphosis,^60^ though this is not always the case.^61^ Studies specifically assessing MeHg and Se across metamorphosis yield inconsistent results; some studies suggest loss of MeHg,^62^ while others note retention of Se^21,63^ and MeHg.^64−66^ Here we show that MeHg retention across life stages is dependent on aqueous Se and dietary MeHg exposure levels, under certain contexts. While there is some evidence for interaction, there is also risk for harm from Se across mayfly life stages given that Se is also toxic to aquatic animals including insects.^9^

### Implications

Adult aquatic insects are important energy subsidies to riparian food webs,^14,20^ as well as important biovectors of aquatic contaminants to terrestrial environments^22,27,28^ when these contaminants are retained across metamorphosis.^19,67,68^ Given the observed elevated MeHg concentrations in adult life stages compared to aquatic larval life stages, high rates of aquatic insect emergence could result in an important aquatic-terrestrial vector of MeHg – irrespective of aqueous Se exposure level and dietary MeHg treatment – and could expose terrestrial predators to this potent neurotoxin. This terrestrial exposure is attributable to the large effect of metamorphosis and molting on increasing MeHg concentrations in mayfly tissue across all aqueous Se exposure levels and MeHg treatments.

We found evidence for Se interaction with MeHg in mayflies, such that elevated Se concentrations reduced MeHg accumulation in mayflies at all life stages when exposed to high MeHg treatment levels. These results support the hypothesis that environmental Se can reduce MeHg bioaccumulation and burden accumulation in aquatic food webs.^9^ However, we also found evidence for a synergistic enhancement of MeHg transfer between life stages with increased aqueous Se exposure levels, particularly at low dietary MeHg treatments. The effect of environmental Se on MeHg uptake, accumulation, and transfer across life stage is highly context-dependent; Se enhanced MeHg uptake and accumulation at low MeHg treatment, but reduced MeHg uptake, accumulation, and transfer at high MeHg treatment. This suggests that the interaction between Hg and Se is not purely based on molar ratios as some literature suggests,^9^ though it could be useful to measure these ratios in future studies conducted at the base of the food web for a comparison to the oft-cited 1:1 Se:Hg molar ratios as protective. Therefore, the interaction between Hg and Se at the base of the food web is highly dependent both upon dietary MeHg levels and organismal life stage.

The idea that Se reduces Hg exposure risk has been promoted as an established, well-characterized phenomenon, with the implication that Se could play a key role in managing Hg risks for the environment.^10,11^ Evidence for Se reducing Hg bioaccumulation, burden accumulation, and biomagnification in aquatic food webs is ambiguous and limited to a few laboratory and field experiments.^9^ Here we found little evidence to support this Se-antagonism hypothesis in a grazing aquatic insect, a potentially important entry pathway for Hg into freshwater food webs. Likewise, we found no evidence that Se limited internal Hg concentrations across life history, when larval insects metamorphosize and disperse to land. This implies that Se has little ameliorative effect on Hg accumulation that would alter Hg risks in either aquatic or terrestrial food webs that depend on aquatic insect subsidies, though the results could be different for higher trophic level organisms. These results run counter to arguments calling for the use of Se as a potential tool to manage Hg exposure risk in aquatic ecosystems. The broader implication of our findings is that experimental support for the widely accepted view that Se can block MeHg uptake in organisms remains limited. Until stronger, mechanistic studies characterize the conditions under which Se limits MeHg uptake in consumers, we should be wary of calls to take management actions such as lowering fish consumption advisories or to amend ecosystems with Se as a mitigation strategy.

## Data Availability

All raw data and associated metadata are available online.^29^

## Supporting Information Available

Additional figure examining the effect of selenium on mayfly methylmercury burden accumulation (Figure S1). Additional table showing the predicted aqueous selenium exposure levels above which the transfer factor equals 1 (Table S1) (PDF)
