# Supplementary material for: Selenium Differentially Influences Methylmercury Retention across Mayfly Life Stages
Source: Environ Sci Technol. 2025 Apr 16;59(16):8201–9. doi: 10.1021/acs.est.5c00338 (PMC12044700; doi:10.1021/acs.est.5c00338)
Supplement: Supplementary file 1 — es5c00338_si_001.pdf [file es5c00338_si_001.pdf]

Title: Selenium differentially influences methylmercury retention across mayfly life stages
Authors: Jacqueline R Gerson,[a*] Rebecca Dorman,[b] Collin Eagles-Smith,[c] David M. Walters[b]
Affiliations:
[a]Cornell University, 111 Wing Dr, Ithaca, NY, 14852 USA, jacqueline.gerson@cornell.edu
[b]U.S. Geological Survey, 4200 E New Haven Rd, Columbia, MO, 65201 USA
[c]U.S. Geological Survey, 777 NW 9th St, Corvallis, OR, 97330 USA
[*]Corresponding author

Acknowledgement: Any use of trade, firm, or product names is for descriptive purposes only and does not imply endorsement by the U.S. Government.

Summary: One page containing an additional figure examining the effect of selenium on mayfly methylmercury burden accumulation (Figure S1) and an additional table showing the predicted aqueous selenium exposure levels above which the transfer factor equals 1 (Table S1).

Effect of Se on MeHg Burden Accumulation

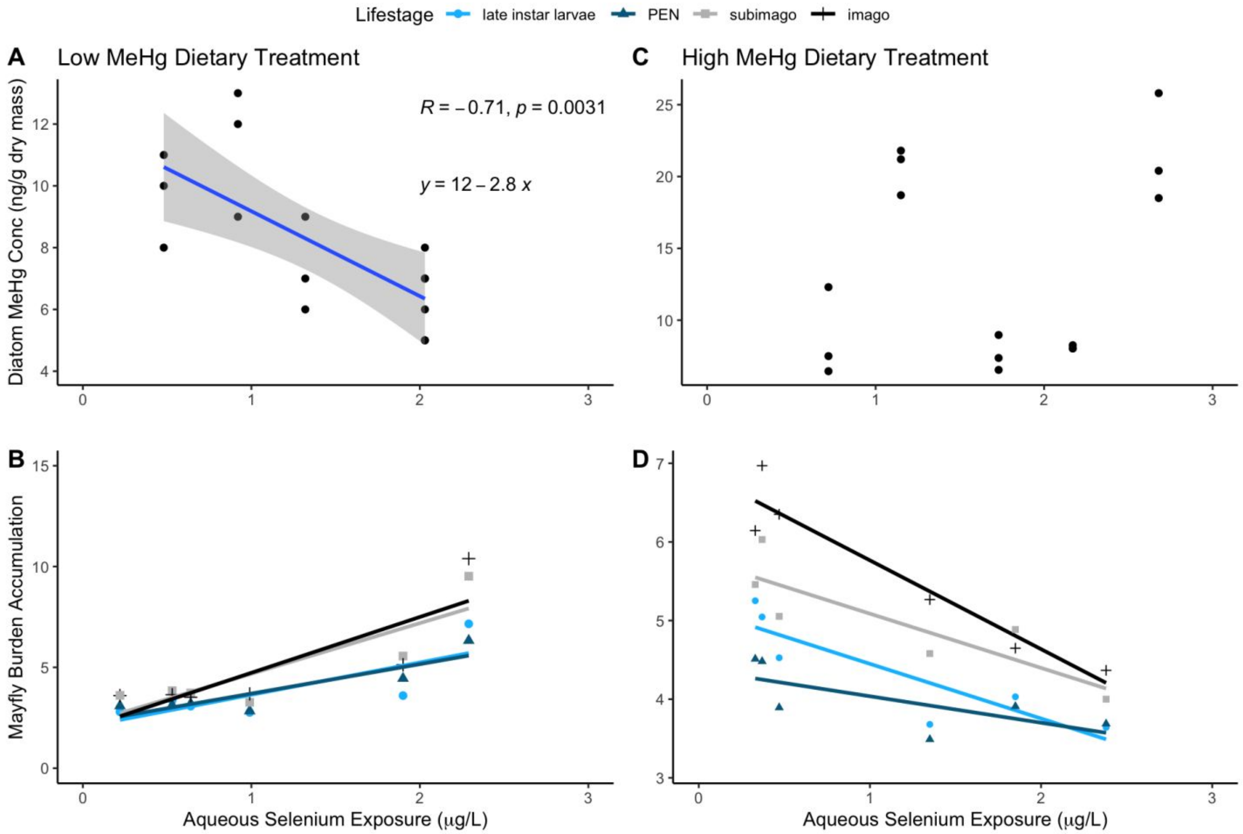

Figure S1: A-B) Relationship between aqueous selenium (Se) exposure treatments and accumulation of methylmercury (MeHg) in diatoms at low (A) and high (B) MeHg dietary treatments, modified from Gerson et al.[1] C-D) Burden accumulation of MeHg in mayflies from diatoms across varying aqueous Se exposures at low (C) and high dietary (D) MeHg dietary treatments. The relationships shown in A and B were used in calculated the biomagnification factors in C and D, respectively. Points are composite values (six to eighteen mayflies) by Se exposure concentration. Linear regression lines are fit for each life stage from full ANCOVA model.

MeHg Transfer between Life Stages

Table S1: Predicted aqueous selenium exposure levels (μg L^{-1}) above which the transfer factor equals 1. Values were calculated using the linear regressions for low and high MeHg dietary treatments, as shown in Figure 2.

Process	Low MeHg Dietary Treatment	High MeHg Dietary Treatment
Elimination	0.37	3.181
Metamorphosis	25.84	5.78
Molting	0.84	4.03
Cumulative	-0.34	23.61

References

1. Gerson, J. R., Dorman, R., Eagles-Smith, C., Bernhardt, E. S. & Walters, D. Lethal impacts of selenium counterbalance the potential reduction in mercury bioaccumulation for freshwater organisms. *Environmental Pollution* **287**, 117293 (2021).
